# Mechanisms and Clinical Implications of the Post-traumatic Stress Disorder (PTSD)–Cardiovascular Disease Link

**DOI:** 10.7759/cureus.100349

**Published:** 2025-12-29

**Authors:** Abubakar I. Sidik, Vladislav V Dontsov, Mikhail G Ruchkin, Nina S Kapieva, Bushra M Alimagomaev, Olga S Epimakhova, Nadiya M Toktarova, Amatuni A Badoyan, Anton N Falchinsky, Noemi D Kirkevich, Anton A Kolyada

**Affiliations:** 1 Department of Cardiovascular Surgery, Peoples' Friendship University of Russia, Moscow, RUS; 2 Department of Cardiothoracic Surgery, Moscow Regional Research and Clinical Institute, Moscow, RUS; 3 Department of Anesthesiology and Intensive Care, Sklifosovsky Institute for Emergency Medicine, Moscow, RUS; 4 Department of Cardiovascular Medicine, Russian University of Medicine (Moscow State University of Medicine and Dentistry), Moscow, RUS; 5 Department of Cardiovascular Medicine, Peoples’ Friendship University of Russia, Moscow, RUS; 6 Department of Cardiovascular Medicine, I.M. Sechenov First Moscow State Medical University, Moscow, RUS; 7 Department of Cardiovascular Surgery, Moscow Regional Research and Clinical Institute, Moscow, RUS; 8 Department of Cardiovascular Medicine, A. S. Puchkov Emergency and Urgent Medical Care Station, Moscow, RUS; 9 Department of Surgery, Federal State Budgetary Institution “Clinical Hospital No. 1” of the Administrative Department of the President of the Russian Federation, Moscow, RUS

**Keywords:** autonomic dysfunction, cardiovascular disease, hypothalamic–pituitary–adrenal axis, mental health and cardiology, post-traumatic stress disorder, veterans health

## Abstract

Post-traumatic stress disorder (PTSD) is increasingly recognized as a significant contributor to cardiovascular morbidity and mortality. The aim of this review was to synthesize current evidence on the biological, behavioral, and clinical mechanisms linking PTSD with cardiovascular disease (CVD) and to highlight clinical implications for screening, prevention, and management. To achieve this, relevant studies were identified through targeted searches of PubMed, Scopus, Web of Science, and Google Scholar using combinations of terms related to PTSD, CVD, autonomic dysfunction, inflammation, endothelial function, and trauma-informed care. Priority was given to original clinical studies, systematic reviews, meta-analyses, and experimental research published between 1990 and 2025.

Findings across large epidemiologic studies show that PTSD is associated with significantly higher rates of hypertension, coronary artery disease, myocardial infarction, stroke, metabolic syndrome, and cardiovascular mortality. Veterans represent the most extensively studied group, with multiple cohorts demonstrating a dose-response relationship between PTSD severity and cardiovascular risk. The review identifies several interconnected biological pathways that explain this association, including chronic sympathetic activation, reduced parasympathetic tone, impaired hypothalamic-pituitary-adrenal axis regulation, systemic inflammation, endothelial dysfunction, platelet hyperreactivity, coagulation abnormalities, oxidative stress, and metabolic disturbances. These mechanisms are further amplified by behavioral factors such as elevated smoking rates, physical inactivity, unhealthy diet, poor sleep, and reduced adherence to cardioprotective medications. Clinical challenges including healthcare avoidance, mistrust of medical environments, and frequent psychiatric comorbidities contribute to delayed diagnosis and poorer outcomes.

Intervention studies suggest that treating PTSD through trauma-focused psychotherapy, selective serotonin reuptake inhibitors, prazosin for sleep symptoms, and structured exercise may improve physiological parameters relevant to cardiovascular risk. Integrated models of care that combine mental health and cardiovascular services, as well as emerging digital tools such as wearables and telepsychiatry, show promise for enhancing monitoring and preventive care.

Overall, current evidence indicates that PTSD is a substantial and modifiable cardiovascular risk factor. Recognition of this relationship supports routine cardiovascular risk assessment in individuals with PTSD and systematic PTSD screening in cardiac care settings. Multidisciplinary, trauma-informed care models may be essential for reducing long-term cardiovascular morbidity in this high-risk population.

## Introduction and background

Post-traumatic stress disorder (PTSD) is a prevalent and debilitating psychiatric condition that arises following exposure to traumatic or life-threatening events. It is characterized by intrusive memories, hyperarousal, avoidance behaviors, and negative alterations in mood and cognition. Globally, lifetime prevalence estimates range from 3% to 8% in the general population, with significantly higher rates among combat veterans, refugees, and survivors of interpersonal violence [1,2]. While PTSD has long been conceptualized as a mental health disorder, increasing evidence over the past two decades highlights its profound systemic effects, particularly its association with cardiovascular disease (CVD) [3,4].

Epidemiological studies consistently demonstrate that individuals with PTSD have significantly higher rates of hypertension, coronary artery disease (CAD), stroke, and cardiovascular mortality compared with the general population. Large veteran cohort analyses further reveal a robust and graded association between PTSD symptom burden and adverse cardiovascular outcomes, underscoring PTSD as an independent and clinically meaningful cardiovascular risk factor [5,6].

The biological and behavioral mechanisms underlying this association are multifactorial. Chronic hyperarousal and persistent activation of the sympathetic nervous system lead to autonomic dysregulation, while dysregulation of the hypothalamic-pituitary-adrenal (HPA) axis contributes to altered cortisol dynamics and enhanced inflammatory responses [7,8]. Elevated circulating levels of inflammatory cytokines such as interleukin-6 (IL-6) and C-reactive protein (CRP), endothelial dysfunction, platelet hyperreactivity, and metabolic disturbances have all been documented in PTSD populations, linking psychological stress to atherosclerotic progression [9,10]. In parallel, behavioral factors such as smoking, physical inactivity, poor diet, sleep disruption, and medication non-adherence exacerbate these biological vulnerabilities [11,12].

Beyond mechanistic understanding, the PTSD-CVD relationship has important clinical implications. PTSD frequently coexists with depression, anxiety, and substance use disorders, compounding cardiovascular risk and complicating treatment [13,14]. Moreover, avoidance behaviors and medical mistrust may delay care-seeking and reduce adherence to preventive therapies, leading to late presentation and worse outcomes [15]. Recognition of this bidirectional relationship has prompted growing advocacy for trauma-informed, multidisciplinary care models that integrate psychiatric and cardiovascular management [16,17].

The aim of this narrative review is to synthesize current evidence on the pathways linking PTSD to CVD in the general population, integrating biological, behavioral, and clinical findings to clarify how trauma-related psychopathology contributes to cardiovascular morbidity and mortality. Additionally, the review seeks to examine emerging implications for screening, prevention, and multidisciplinary management to guide clinical practice and future research.

## Review

Methods

Relevant literature was identified through targeted searches of PubMed, Scopus, Web of Science, and Google Scholar using combinations of terms related to PTSD, CVD, hypertension, CAD, autonomic dysfunction, inflammation, and trauma-informed care.

The search focused on articles published between 1990 and 2025 and prioritized human clinical research, epidemiological cohort studies, longitudinal analyses, mechanistic studies, and high-quality systematic reviews or meta-analyses that examined biological, behavioral, or clinical pathways linking PTSD with CVD. Studies were included if they met one or more of the following criteria: (1) evaluated PTSD as an exposure variable for cardiovascular outcomes; (2) investigated biological or behavioral mechanisms relevant to cardiovascular risk; or (3) presented clinical implications or intervention data applicable to PTSD populations.

Animal studies, case reports, single-patient observations, and papers unrelated to the PTSD-cardiovascular association were excluded unless they provided mechanistic insight not available from human data. Reference lists of key publications were also scanned to identify additional eligible studies.

Because this review is narrative in scope, no formal quality scoring tool or PRISMA flow diagram was applied. However, when integrating study findings, particular consideration was given to sample size, study design, generalizability, and risk of confounding. Observational study limitations, including self-report bias, diagnostic heterogeneity, and lack of randomized evidence, were acknowledged during synthesis.

Epidemiology of the PTSD-CVD link

A substantial body of epidemiological research demonstrates that PTSD is a significant and independent risk factor for major CVD outcomes. Large-scale cohort studies, population-based surveys, and veteran health databases consistently show higher rates of hypertension, CAD, myocardial infarction (MI), stroke, metabolic syndrome, and cardiovascular mortality among individuals with PTSD compared with trauma-exposed or non-trauma-exposed controls. These associations persist even after adjusting for traditional risk factors such as smoking, obesity, diabetes, and socioeconomic status [6,18].

Meta-analytic findings reinforce this elevated risk. PTSD is associated with approximately a 55% increased risk of incident coronary heart disease and nearly double the risk of cardiovascular-related mortality compared with the general population. Epidemiological studies also highlight a dose-response pattern in which greater PTSD symptom severity correlates with higher cardiovascular risk, suggesting a graded relationship between psychological trauma and physical disease burden [19,20].

Among all populations studied, military veterans represent the most extensively researched group, largely because of their high levels of trauma exposure and comprehensive longitudinal health records. Multiple veteran cohorts (from World War II to Afghanistan) demonstrate markedly elevated rates of both subclinical and clinical cardiovascular pathology. These include higher prevalence of conduction defects, early atherosclerosis, heart failure, and increased cardiovascular mortality. The epidemiological patterns documented in veteran samples are robust and consistent across decades, geographic regions, and methodological approaches [21-23].

Importantly, the increased cardiovascular risk is not limited to older veterans with long-standing PTSD. Contemporary data from Iraq and Afghanistan veterans demonstrate that even younger adults with PTSD exhibit significantly higher incidence of coronary heart disease, stroke, and transient ischemic attacks, underscoring the early onset and potentially long-term trajectory of CVD risk linked to trauma exposure [22,24,25]. Table 1 summarizes findings from major veteran cohort studies spanning World War II, the Vietnam era, and post-9/11 service members. These studies consistently demonstrate elevated rates of CAD, MI, stroke, and cardiovascular mortality among veterans with PTSD, often in a dose-dependent manner.

**Table 1:** Epidemiological Evidence Linking PTSD to Cardiovascular Disease Among Military Veterans PTSD, Post-Traumatic Stress Disorder; CVD, Cardiovascular Disease; CAD, Coronary Artery Disease; CHD, Coronary Heart Disease; MI, Myocardial Infarction; IHD, Ischemic Heart Disease; CHF, Congestive Heart Failure; PVD, Peripheral Vascular Disease; TIA, Transient Ischemic Attack; MACE, Major Adverse Cardiovascular Events; WWII, World War II

Author/Year	Design Type	Setting	Participant Characteristics	Cohort Size	Cardiovascular Findings (% Increases)
Falger et al., 1992 [21]	Clinical assessment	Netherlands	WWII Resistance veterans vs controls	147 + controls	PTSD cases showed more cardiac symptoms; ~10% reported MI.
Boscarino, 1997 [22]	Interview-based observational	USA	Vietnam Army veterans	1,399	Circulatory disorders ~62% more common with PTSD.
Boscarino & Chang, 1999 [25]	Medical & psychiatric evaluation	USA	Vietnam theatre vs era veterans	2,490 + 1,972	Conduction defects ~180% higher; infarction signs ~340% higher in PTSD cases.
Schnurr et al., 2000 [26]	Prospective follow-up	USA	WWII & Korean War veterans	605	Arterial disorders ~30% more likely with higher PTSD symptoms.
Kang et al., 2006 [27]	Retrospective cohort	USA	WWII prisoners of war	19,442	IHD ~19% higher; MI ~11% higher among those with PTSD.
Boscarino, 2006 [28]	Mortality cohort	USA	Vietnam veterans	15,288	Cardiovascular mortality ~70% higher.
Kubzansky et al., 2007 [29]	Prospective cohort	USA	Older male veterans	1,002	MI ~30% higher; CHD ~21% higher with PTSD symptoms.
Boscarino, 2008 [24]	Cohort study	USA	Vietnam veterans <65 yrs	4,328	Heart-disease mortality ~125% higher with PTSD.
Ahmadi et al., 2011 [18]	Cohort	USA	Veterans without CAD	637	Coronary atherosclerosis ~59% more likely with PTSD.
Scherrer et al., 2010 [30]	Retrospective cohort	USA	US Veterans	355,999	MI ~39% higher among PTSD patients.
Beristianos et al., 2016 [31]	Retrospective cohort	USA	Veterans ≥55	138,341	CVD ~45% higher; CHF ~26% higher; MI ~49% higher; PVD ~35% higher.
Crum-Cianflone et al., 2017 [23]	Prospective cohort	USA	Iraq/Afghanistan veterans	60,025	CHD ~66% more likely among PTSD cases.
Rosman et al., 2019 [32]	Cohort	USA	Discharged service members	987,855	TIA ~61% higher; stroke ~36% higher.
El-Solh et al., 2022 [33]	Retrospective cohort	USA	Veterans with PTSD or insomnia	19,080	MACE ~67% more frequent with PTSD.

Biological mechanisms

Autonomic Dysregulation

People with PTSD frequently show hyperarousal accompanied by increased sympathetic drive and reduced parasympathetic control. Meta-analytic evidence demonstrates that individuals with PTSD have significantly lower heart rate variability (HRV) at rest and during stress, indicating vagal withdrawal and relative sympathetic dominance [34]. Reviews of mechanistic studies further describe elevated resting heart rate, greater catecholamine release, and altered sympathovagal balance in PTSD, all of which contribute to cardiovascular risk [3]. Clinical and experimental studies consistently confirm these findings, showing a higher heart rate and reduced HRV in PTSD, reflecting impaired autonomic flexibility [35].

Ambulatory studies provide additional support for this dysregulation in daily life. For example, daily monitoring of World Trade Center responders revealed that increases in PTSD symptoms were associated with concurrent decreases in HRV, suggesting moment-to-moment coupling between psychological distress and autonomic control [36]. Baroreflex dysfunction appears to be another key feature of PTSD-related autonomic disturbance. In a physiological study of combat veterans by Park et al., those with PTSD exhibited exaggerated sympathetic responses to mental stress and impaired baroreflex sensitivity compared to healthy controls [37]. Veterans with PTSD exhibit exaggerated muscle sympathetic nerve activity and heart rate responses during virtual reality combat exposure, indicating heightened sympathetic reactivity [37]. Evidence from interventional studies further supports the role of modifiable autonomic mechanisms. Device-guided slow breathing, which enhances vagal tone, acutely improves baroreflex function and reduces sympathetic nerve activity in patients with PTSD [38,39].

HPA Axis Dysfunction

Alterations in the HPA axis are a central biological feature of PTSD. Numerous studies have shown that individuals with PTSD tend to have lower baseline cortisol levels than trauma-exposed or healthy controls, suggesting enhanced glucocorticoid receptor sensitivity and stronger negative feedback inhibition of the HPA axis [40,41]. This pattern is accompanied by a flattened diurnal cortisol slope, where cortisol secretion shows a reduced decline from morning to evening. Such blunted rhythmicity indicates disrupted circadian regulation and an impaired ability to modulate the stress response across the day [42].

Another consistent finding is corticotropin-releasing hormone (CRH) hypersecretion, reflecting excessive hypothalamic drive even when circulating cortisol levels are low. Elevated CRH concentrations have been documented in the cerebrospinal fluid of individuals with PTSD, consistent with central HPA overactivation and chronic arousal [43]. This imbalance between central CRH activation and peripheral cortisol suppression may help explain the paradoxical coexistence of anxiety, inflammation, and metabolic dysregulation in PTSD. Sustained HPA axis disruption can contribute to endothelial dysfunction, hypertension, and atherosclerosis, thereby linking chronic stress to cardiovascular risk [40,44].

**Figure 1:**
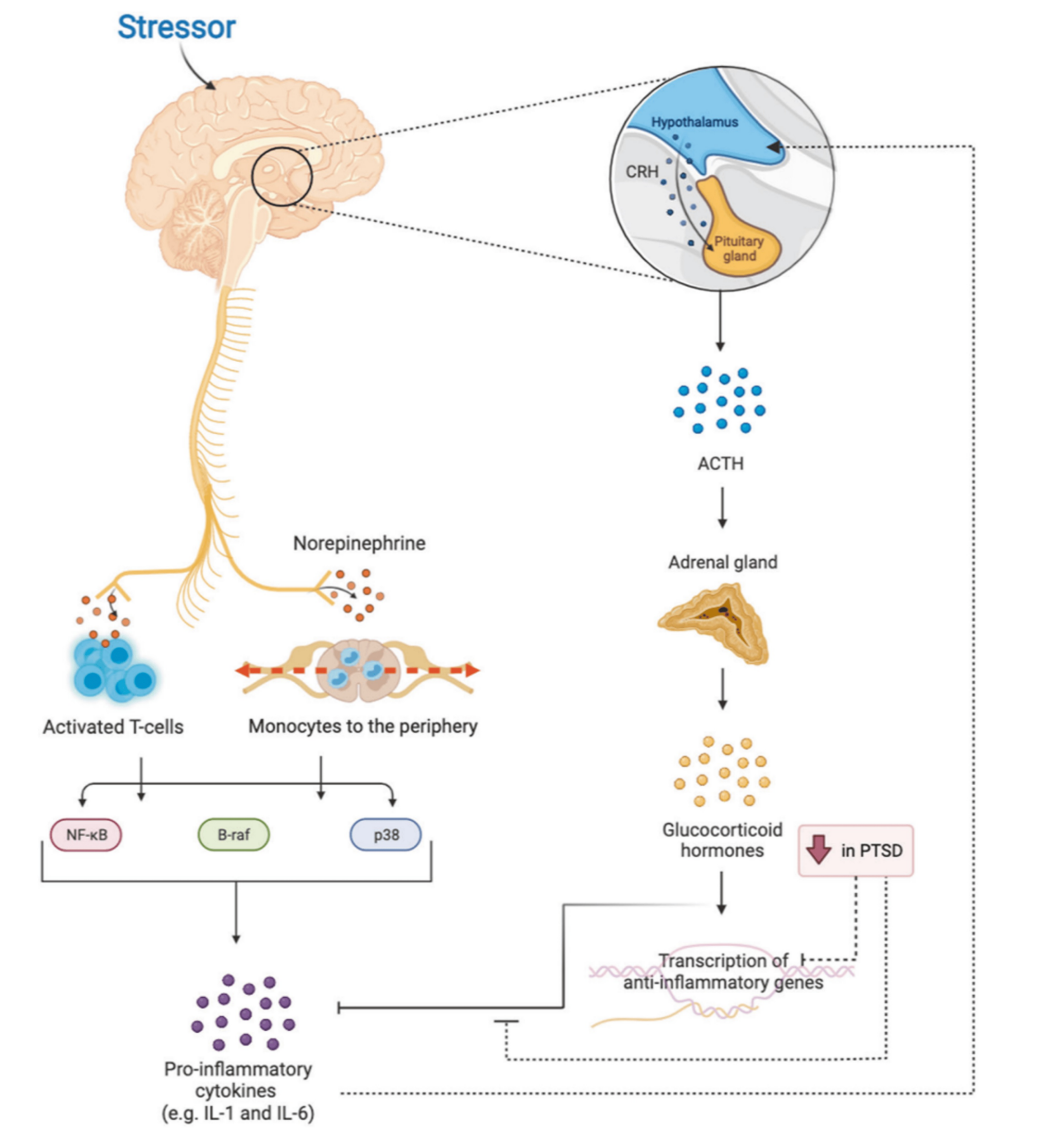
Relationship between the Hypothalamic–Pituitary–Adrenal (HPA) Axis, Sympathetic Nervous System (SNS), and Inflammation in PTSD Stress exposure stimulates the SNS and HPA axis, leading to norepinephrine release, activation of pro-inflammatory signaling pathways (NF-κB, B-raf, p38), and impaired glucocorticoid feedback that sustains inflammation. Reprinted under the terms of the Creative Commons Attribution 4.0 International License from Katrinli et al. [45] PTSD, Post-traumatic Stress Disorder; ACTH, Adrenocorticotropic Hormone

The interaction between the HPA axis and the sympathetic nervous system (SNS) provides a key biological link between stress-related psychopathology and inflammation. As illustrated in Figure 1, stress exposure activates both systems, resulting in catecholamine release, monocyte and T-cell activation, and production of pro-inflammatory cytokines such as IL-1 and IL-6. Simultaneously, reduced glucocorticoid sensitivity in PTSD diminishes anti-inflammatory gene transcription, sustaining chronic inflammation.

Inflammation and Immune Activation

PTSD has been increasingly recognized as a condition involving chronic low-grade inflammation and immune dysregulation, which may contribute to elevated cardiovascular risk. Meta-analytic evidence consistently demonstrates that individuals with PTSD have higher circulating concentrations of inflammatory biomarkers, including CRP, IL-6, and tumor necrosis factor-alpha, compared with trauma-exposed or healthy control groups [9,46]. These pro-inflammatory cytokines are associated with endothelial dysfunction, oxidative stress, and acceleration of atherosclerotic processes, providing a mechanistic bridge between chronic psychological stress and cardiovascular disease.

Beyond the classical inflammatory markers, recent research has explored the role of innate immune activation through the NOD-like receptor pyrin domain-containing 3 (NLRP3) inflammasome. Activation of NLRP3 promotes the maturation and secretion of IL-1β and IL-18, two cytokines implicated in both neuroinflammation and vascular pathology. Preclinical studies using rodent models of PTSD have shown that exposure to traumatic stress can activate NLRP3 signaling pathways in the hippocampus and amygdala, leading to neuroinflammation and anxiety-like behaviors; pharmacologic inhibition of NLRP3 attenuates these effects [45,47]. Though human evidence is limited, inflammasome activation has emerged as a plausible pathway connecting trauma-related stress, immune activation, and cardiovascular dysfunction.

Endothelial Dysfunction

Endothelial dysfunction is a key early event in the progression of CVD, and growing evidence indicates that individuals with PTSD have significant impairments in vascular endothelial function. A pivotal study among veterans demonstrated that those with current PTSD had markedly lower brachial artery flow-mediated dilation (FMD) compared with those without PTSD, suggesting compromised endothelium-dependent vasodilation [48]. The study also identified reductions in biomarkers of nitric oxide (NO) synthesis in the PTSD group, implicating trauma-related stress in diminished NO bioavailability [48].

Nitric oxide is essential for vascular homeostasis, as it promotes vasodilation, inhibits platelet aggregation, suppresses smooth-muscle proliferation, and prevents leukocyte adhesion to the endothelium. Reduced NO bioavailability in PTSD likely arises from a combination of chronic sympathetic activation, oxidative stress, and systemic inflammation, which together impair endothelial nitric oxide synthase activity and enhance NO degradation [49,50]. Experimental studies in animal models of PTSD further support these mechanisms, showing that stress exposure leads to reduced NO responsiveness, elevated oxidative stress within endothelial tissue, and impaired vasodilatory capacity [50].

Flow-mediated dilation serves as a noninvasive ultrasound measure of conduit artery endothelial function and has been used extensively in PTSD research. In addition to findings from veteran populations, recent studies involving trauma-exposed women have shown that greater PTSD symptom severity is independently associated with lower FMD, even after adjusting for traditional cardiovascular risk factors, suggesting a graded relationship between trauma-related stress and vascular dysfunction [51]. The reduction in FMD reflects a diminished ability of the artery to dilate in response to shear stress, a key indicator of NO-mediated endothelial responsiveness and an established predictor of future cardiovascular events.

Coagulation and Thrombosis

Disturbances in coagulation and thrombotic pathways have been increasingly implicated in the link between PTSD and CVD. Chronic psychological stress and sustained sympathetic activation in PTSD appear to promote a pro-thrombotic state characterized by platelet hyperreactivity and increased circulating coagulation factors such as fibrinogen and D-dimer.

Platelet hyperreactivity: Patients with PTSD demonstrate heightened platelet activity, which contributes to an increased risk of thrombosis and atherosclerotic progression. In one controlled study, war veterans with PTSD showed exaggerated platelet aggregation and increased expression of activation markers compared with non-PTSD controls, consistent with a hyperreactive platelet phenotype [52]. Stress-induced catecholamine surges and altered serotonergic signaling are thought to enhance platelet activation and aggregation [53]. These mechanisms align with the broader evidence that sympathetic overdrive in PTSD contributes to vascular injury and thrombus formation.

Elevated fibrinogen and D-dimer: In addition to platelet changes, abnormalities in coagulation biomarkers have been documented in PTSD. Chronic stress and PTSD are associated with elevated plasma fibrinogen levels, an important clotting factor that increases blood viscosity and enhances the risk of vascular occlusion [54]. Elevated D-dimer, a fibrin degradation product, has also been observed in PTSD patients, reflecting increased thrombin generation and fibrinolytic activity [55]. These abnormalities indicate ongoing activation of the coagulation system and may represent a biological link between PTSD and heightened cardiovascular morbidity.

Metabolic Dysregulation

There is a growing body of evidence that individuals with PTSD are at elevated risk of metabolic disturbances, including insulin resistance, metabolic syndrome, and dyslipidemia. One key longitudinal study found that PTSD was independently associated with increased incidence of insulin resistance and metabolic syndrome over follow‐up [56]. More specifically, in a large cross‐sectional study of trauma‐exposed civilians, PTSD was associated with 1.53 times higher odds of metabolic syndrome among women, and PTSD symptom severity showed a graded association with metabolic risk [57].

With regard to dyslipidemia, a recent meta‐analysis of lipid profiles in PTSD found that patients with PTSD had significantly higher levels of total cholesterol, low‐density lipoprotein (LDL) and triglycerides, along with lower high‐density lipoprotein (HDL) compared with healthy controls [58]. These lipid abnormalities, combined with insulin resistance and central adiposity, form core components of the metabolic syndrome cluster, which is itself a strong predictor of CVD.

Mechanistically, chronic stress and PTSD contribute to metabolic dysregulation through multiple pathways, dysregulated HPA axis and cortisol signaling, autonomic imbalance with sympathetic overactivity, pro‐inflammatory immune activation, behavioral risk patterns (poor diet, sedentary lifestyle, sleep disturbance), and adverse effects of psychotropic medications. These pathways lead to visceral adiposity, impaired glucose disposal, increased hepatic lipid synthesis, and impaired lipid clearance, promoting insulin resistance and atherogenic dyslipidemia. To provide a concise visual overview of the discussed mechanisms, the key biological pathways through which PTSD may contribute to cardiovascular disease are summarized in Table 2.

**Table 2:** Biological Mechanisms Linking PTSD to Cardiovascular Disease PTSD, Post-traumatic Stress Disorder; IL-6, Interleukin 6; CAD, Coronary Artery Disease; CVD, Cardiovascular Disease; MI, Myocardial Infarction

Mechanism	Core Physiological Features	Proposed Pathway to Cardiovascular Disease	Representative Cardiovascular Outcomes	Key References
Autonomic Nervous System Dysregulation	Chronic sympathetic activation; reduced parasympathetic tone; elevated resting heart rate; impaired HRV	Persistent catecholamine surge increases vascular tone, myocardial workload, arrhythmogenic potential, and BP variability	Hypertension, arrhythmia, myocardial ischemia, sudden cardiac death	Edmondson & Cohen [3]; Schneider & Schwerdtfeger [34]; Dennis et al. [35]; Park et al. [37]
HPA Axis Dysregulation & Cortisol Abnormalities	Blunted diurnal cortisol curve; glucocorticoid resistance; impaired feedback suppression	Promotes visceral adiposity, insulin resistance, dyslipidemia, and endothelial inflammation, accelerating atherosclerosis	Metabolic syndrome, coronary artery disease, accelerated atherosclerosis	Yehuda & Seckl [40]; Lawrence & Scofield [8]; Miller et al. [42]; Dunlop & Wong [44]
Systemic Inflammation & Immune Activation	Elevated CRP, IL-6, TNF-α; monocyte activation; chronic low-grade inflammation	Inflammatory endothelial injury facilitates plaque formation and rupture	Coronary heart disease, stroke, peripheral vascular disease	Passos et al. [9]; Yang & Jiang [46]; Katrinli et al. [45]
Platelet Hyperreactivity & Coagulation Changes	Increased platelet aggregation; elevated fibrinogen; reduced fibrinolysis	Heightened thrombogenic state raises risk of vessel occlusion and ischemic events	MI, thromboembolism, stroke	Vidović et al. [52]; Dietrich-Muszalska & Wachowicz [53]; von Känel [54]
Endothelial Dysfunction	Reduced nitric oxide bioavailability; impaired vasodilation; pro-oxidative stress	Promotes vascular stiffness, impaired coronary perfusion, and early atherogenesis	CAD, heart failure with preserved EF, microvascular angina	Grenon et al. [48]; Oosthuizen et al. [49]; Sher et al. [50]
Sleep Disturbance & Circadian Disruption	Fragmented sleep architecture; REM disruption; circadian misalignment	Worsens hypertension, metabolic dysregulation, autonomic imbalance, and inflammation	Hypertension, diabetes, obesity-related CVD	Meinhausen et al. [12]; Solh et al. [33]
Metabolic & Adiposity Pathways	Elevated BMI, visceral fat accumulation, dyslipidemia	Obesity-associated inflammatory pathways accelerate vascular injury	Atherosclerotic CVD, diabetes-related cardiac disease	Hoerster et al. [11]; Kinder et al. [20]; Meinhausen et al. [12]
Oxidative Stress	Excess reactive oxygen species; mitochondrial strain	Damages vascular endothelium and myocardium	Ischemic injury, cardiomyopathy	Oosthuizen et al. [49]; Sher et al. [50]
Behaviorally Mediated Physiological Change	Stress-driven smoking, alcohol, inactivity, poor diet	Amplifies biological pathology and inflames vascular risk beyond direct PTSD effects	MI, stroke, heart failure	Hoerster et al. [11]; Meinhausen et al. [12]

Behavioral and lifestyle pathways

Smoking and Substance Use

Individuals with PTSD have significantly higher rates of tobacco use and substance abuse compared with trauma-exposed or general population groups. Smoking prevalence in people with PTSD is estimated to be two to four times higher than in those without the disorder, with PTSD also linked to greater nicotine dependence and reduced cessation success [59,60]. In a large clinical sample, trauma exposure and PTSD were independently associated with increased smoking frequency and severity of nicotine dependence, even after controlling for depression and anxiety [60].

These behavioral patterns have major cardiovascular implications. Chronic smoking promotes endothelial injury, oxidative stress, inflammation, and platelet activation, which accelerate atherosclerosis and CVD progression [6,61]. Among individuals with PTSD, smoking may also intensify sympathetic activation and catecholamine surges, compounding the biological stress burden that underlies their elevated cardiovascular risk [6].

Substance use, including alcohol, cannabis, and stimulants, frequently co-occurs with PTSD and further contributes to poor cardiovascular outcomes. Substance use disorders are associated with increased impulsivity, poor treatment adherence, and higher rates of hypertension, arrhythmia, and metabolic dysfunction [6,62]. Alcohol and stimulant use also exacerbate sleep disturbance and sedentary behavior, amplifying modifiable cardiovascular risk factors in PTSD populations.

Physical Inactivity

Physical inactivity is a major behavioral pathway linking PTSD to increased CVD risk. Longitudinal cohort studies show that individuals with PTSD are more likely to engage in lower levels of physical activity over time compared with trauma-exposed individuals without PTSD. In a 20-year prospective study of U.S. veterans, higher PTSD symptom levels were associated with a steeper decline in leisure-time physical activity, which may partially account for the higher burden of cardiovascular morbidity observed in this population [63].

Physical inactivity contributes to cardiovascular risk through several mechanisms, including increased adiposity, insulin resistance, dyslipidemia, elevated blood pressure, and systemic inflammation [64]. In people with PTSD, these effects may be compounded by neuroendocrine dysregulation, autonomic imbalance, and behavioral avoidance patterns, leading to a cycle of sedentary behavior and declining physical health. Evidence from population-based studies suggests that physical inactivity significantly mediates the relationship between PTSD and incident CAD, even after controlling for smoking, alcohol use, and other lifestyle factors [65].

Regular physical activity improves cardiovascular outcomes and may counteract many of the biological alterations associated with PTSD. Exercise enhances autonomic balance, improves endothelial function, lowers inflammation, and reduces metabolic risk factors. Integrating structured, trauma-informed exercise interventions into PTSD treatment and CVD prevention programs could therefore play a vital role in mitigating cardiovascular risk among affected individuals [64,65].

Poor Diet and Obesity

Individuals with PTSD frequently demonstrate unhealthy dietary habits and higher rates of obesity, both of which substantially contribute to CVD risk. A meta-analysis of 29 studies found that compared with individuals without PTSD, those with PTSD were less likely to maintain healthy eating behaviors, more likely to be obese, and more likely to smoke [66]. Similarly, a large national cohort study from Norway reported that PTSD was associated with a significantly increased hazard of developing obesity and related cardiometabolic disorders, even after adjusting for confounders such as age, sex, and socioeconomic status [67].

Unhealthy eating patterns among individuals with PTSD often include higher consumption of calorie-dense, processed foods, refined carbohydrates, and saturated fats, alongside lower intake of fruits, vegetables, and whole grains [68]. These patterns may arise as maladaptive coping mechanisms to chronic stress or as a result of a dysregulated HPA axis and reward pathway function, leading to increased appetite and cravings for high-fat and high-sugar foods. Neuroendocrine changes associated with PTSD, such as elevated cortisol and sympathetic nervous system activation, can also promote visceral adiposity and metabolic dysfunction.

Obesity acts as a major mediator in the pathway linking PTSD to CVD, contributing to insulin resistance, dyslipidemia, hypertension, and systemic inflammation [69]. Evidence from longitudinal studies supports this relationship, showing that the association between PTSD and new-onset diabetes is partly explained by obesity [69].

Sleep Disturbances (Insomnia, Nightmares) 

Sleep disturbances are among the most prevalent behavioral and lifestyle pathways linking PTSD with CVD. Insomnia, characterized by difficulty initiating or maintaining sleep, and recurrent nightmares are experienced by up to 70-90% of individuals with PTSD [70].

These sleep-related problems are associated with heightened physiological stress responses, including increased SNS activity, elevated catecholamine levels, inflammation, and reduced HRV, all of which are established mechanisms contributing to cardiovascular risk [71,72]. Individuals with PTSD and chronic sleep disturbances often exhibit elevated resting heart rate, impaired autonomic recovery following stress, and higher circulating inflammatory cytokines, pathways that are directly implicated in endothelial dysfunction and atherogenesis [71,72].

Longitudinal studies indicate that sleep disturbances may both result from trauma and predict future CVD events. Individuals with pre-existing insomnia or frequent nightmares have higher risks of developing hypertension, CAD, and stroke, even after controlling for traditional risk factors [72,73]. In addition, sleep fragmentation and poor sleep efficiency have been shown to contribute to increased arterial stiffness and subclinical atherosclerosis in PTSD populations [72].

Addressing insomnia and nightmares in PTSD is crucial for cardiovascular prevention. Trauma-informed sleep interventions such as cognitive behavioral therapy for insomnia and imagery rehearsal therapy have been shown to improve sleep quality, reduce PTSD symptom severity, and improve cardiovascular biomarkers, including heart rate variability and inflammatory profiles [72,73].

Medication Non-adherence (Beta-blockers, Statins)

Medication non-adherence is a significant behavioral pathway through which PTSD contributes to increased CVD risk. Patients with PTSD have higher rates of non-adherence to essential cardiovascular medications, including beta-blockers and statins. In one study of hypertensive patients, those with PTSD were nearly three times more likely to be non-adherent to antihypertensive therapy compared with those without PTSD, even after adjusting for depression and sociodemographic factors [74].

Non-adherence diminishes the benefits of evidence-based pharmacotherapy. Beta-blockers mitigate sympathetic overactivation, reduce heart rate and blood pressure, and improve post-myocardial infarction survival, while statins lower LDL cholesterol, stabilize atherosclerotic plaques, and prevent recurrent cardiovascular events. Studies have consistently shown that poor adherence to these medications leads to increased rates of MI, stroke, heart failure, and mortality [75,76].

Multiple psychological and behavioral mechanisms contribute to medication non-adherence in PTSD, including avoidance, cognitive dysfunction, hyperarousal, and disrupted daily routines. Sleep disturbance, emotional numbing, and comorbid depression may also impair self-care and medication consistency. Negative beliefs about medications and mistrust of medical professionals can further reduce adherence. These factors collectively undermine long-term secondary prevention and worsen cardiovascular prognosis [74,76].

Addressing medication non-adherence should be a core component of cardiovascular risk management in patients with PTSD. Evidence supports interventions such as simplified dosing regimens, pharmacist-led counseling, digital adherence aids, and trauma-informed communication approaches to improve adherence and reduce CVD risk [75,76]. To complement the biological mechanisms described above, Table 3 summarizes the key behavioral and lifestyle factors through which PTSD may further contribute to cardiovascular risk.

**Table 3:** Behavioral and Lifestyle Mediators Linking PTSD to Cardiovascular Disease PTSD, Post-traumatic Stress Disorder; CAD, Coronary Artery Disease; CVD, Cardiovascular Disease; MI, Myocardial Infarction

Behavioral/Lifestyle Factor	PTSD-Related Features	Mechanistic Contribution to Cardiovascular Risk	Associated Cardiovascular Outcomes	Key References
Smoking & Nicotine Dependence	Increased smoking initiation, higher consumption rates, reduced cessation success	Promotes endothelial injury, oxidative stress, thrombosis, dyslipidemia, and sympathetic activation	Coronary artery disease, stroke, and peripheral vascular disease	Hoerster et al. [11]; Edmondson et al. [19]; Crum-Cianflone et al. [23]
Physical Inactivity & Sedentary Lifestyle	Avoidance behaviors, fatigue, anhedonia, impaired motivation	Reduces cardiorespiratory fitness, increases adiposity, promotes insulin resistance and hypertension	Obesity-related CVD, heart failure, CAD, and diabetes	Edmondson et al. [19]
Poor Diet/Unhealthy Eating Patterns	Emotional eating, irregular intake, processed food consumption	Elevates cholesterol, blood glucose, adiposity, and inflammatory pathways	Metabolic syndrome, diabetes, and atherosclerosis	Hoerster et al. [11]; Kinder et al. [20]
Alcohol Misuse/Substance Use	Maladaptive coping, self-medication behaviors	Raises BP, triglycerides, arrhythmogenic substrate, oxidative stress	Hypertension, cardiomyopathy, and arrhythmia	Gould et al. [14]; Edmondson & Cohen [3]
Medication Non-adherence	Avoidance, mistrust, executive dysfunction, cognitive symptoms	Reduces control of blood pressure, diabetes, lipids, and platelet activity; worsens treatment outcomes	Recurrent MI, uncontrolled hypertension, and HF exacerbation	Hitch et al. [15]
Sleep Disturbance/ Insomnia/OSA Risk	Nightmares, hyperarousal, fragmented sleep	Raises BP, systemic inflammation, autonomic dysregulation, metabolic dysfunction	Hypertension, stroke, arrhythmia, and diabetes	Meinhausen et al. [12]; El-Solh et al. [33]
Stress-Related Eating or Weight Change	Weight gain or loss linked to chronic stress hormones	Alters metabolic profile, increases visceral fat and inflammatory burden	Atherosclerotic CVD and obesity-related disease	Roer et al. [67]; Masodkar et al. [68]
Lower Healthcare Engagement	Avoidance of clinicians and procedures	Delays detection and management of risk factors	More advanced CAD at diagnosis and preventable events	Hitch et al. [15]; Yehuda et al. [41]

Clinical and treatment-related factors

Psychotropic Medications

Psychotropic medications commonly used in patients with PTSD can influence cardiovascular risk through multiple mechanisms, both beneficial and adverse.

Cardioprotective vs. QT prolongation of antidepressants: Selective serotonin reuptake inhibitors (SSRIs) and serotonin-norepinephrine reuptake inhibitors (SNRIs) are first-line treatments for PTSD and comorbid depression. These agents may indirectly lower cardiovascular risk by alleviating depressive and anxiety symptoms, which are independently associated with adverse cardiovascular outcomes. However, some SSRIs, notably citalopram and escitalopram, can modestly prolong the QT interval, raising the risk of arrhythmias in susceptible patients [77,78]. Evidence from large observational studies suggests that antidepressant use overall does not significantly increase the risk of coronary heart disease (CHD) and may even confer modest cardioprotective effects when used appropriately [79]. Careful cardiac monitoring and dose adjustment are recommended in patients with preexisting heart disease or those on other QT-prolonging agents.

Metabolic syndrome risk of antipsychotics: Second-generation antipsychotic medications, occasionally prescribed as adjunctive therapies for treatment-resistant PTSD or comorbid psychotic symptoms, are associated with metabolic disturbances such as weight gain, dyslipidemia, insulin resistance, and hypertension [80]. These adverse effects contribute to the development of metabolic syndrome and significantly elevate cardiovascular risk. The degree of risk varies across agents: clozapine and olanzapine pose the highest risk, whereas aripiprazole and ziprasidone have a relatively favorable metabolic profile. Regular monitoring of weight, glucose, and lipid parameters is essential for early detection and management of metabolic complications [80].

Blood pressure effects of Prazosin: Prazosin, an alpha-1 adrenergic receptor antagonist, is frequently used for PTSD-related nightmares and sleep disturbances. It lowers systemic vascular resistance and blood pressure, which can be beneficial in hypertensive patients. However, it may also cause orthostatic hypotension, dizziness, or syncope, particularly during initial titration or when combined with other antihypertensive agents [81]. Monitoring of blood pressure and gradual dose adjustments are important to balance efficacy and safety.

Comorbid Psychiatric Conditions

Comorbid psychiatric disorders, particularly depression, anxiety, and substance use disorders, substantially contribute to CVD risk in individuals with PTSD. Depression is one of the most frequent comorbidities in PTSD and has been independently associated with an increased incidence of CAD, MI, and mortality [82]. Anxiety disorders and substance use disorders similarly heighten cardiovascular risk, creating a compounding effect when they coexist with PTSD [65]. Epidemiological evidence underscores this relationship. A national cohort study in Norway demonstrated that when depression and anxiety were accounted for, the association between PTSD and cardiometabolic disease, including hypertension, obesity, and diabetes, was reduced but remained significant, suggesting that comorbid psychiatric conditions mediate but do not fully explain the PTSD-CVD connection [67].

Mechanistically, these comorbidities contribute to CVD through behavioral and physiological pathways. Depression and anxiety are associated with elevated inflammatory markers, increased sympathetic activity, and reduced HRV, all of which promote atherogenesis and cardiac dysfunction [82]. They also correlate with unhealthy behaviors such as physical inactivity, smoking, and poor diet, which further elevate cardiovascular risk. Substance use disorders add an additional layer of complexity by inducing direct cardiotoxic effects such as stimulant-induced arrhythmias or alcohol-related cardiomyopathy and by impairing adherence to medical therapies [65].

Clinically, recognizing and addressing comorbid psychiatric conditions is essential for reducing CVD risk in patients with PTSD. Integrated care approaches that include screening and treatment for depression, anxiety, and substance use disorders may improve both mental and cardiovascular outcomes. Early interventions targeting mood, stress regulation, and substance use can play a critical role in mitigating long-term cardiovascular complications in PTSD populations.

Healthcare Avoidance and Delayed Diagnosis

Healthcare avoidance and delayed diagnosis are significant contributors to adverse cardiovascular outcomes among individuals with PTSD. Avoidance behaviors, a core feature of PTSD, can extend beyond trauma-related contexts into medical care, leading to underutilization of preventive health services, reluctance to seek treatment, and delayed presentation for physical symptoms [83].

Patients with PTSD frequently report mistrust of healthcare providers, anxiety in clinical environments, and fear of retraumatization, which can result in reduced engagement with routine medical visits and cardiovascular screenings [41]. This avoidance has serious implications for CVD management. Among U.S. veterans, PTSD has been associated with lower rates of follow-up after abnormal cardiovascular test results and reduced adherence to primary care appointments, contributing to more advanced disease at diagnosis and poorer long-term outcomes [19,84].

In addition, the overlapping symptoms of PTSD and CVD, such as chest pain, palpitations, and dyspnea, can complicate diagnosis. These symptoms are sometimes misattributed to anxiety or panic, delaying appropriate cardiac evaluation and treatment [19,41]. Comorbid depression and substance use further exacerbate help-seeking delays and reduce adherence to medical advice.

Addressing healthcare avoidance requires a trauma-informed approach that integrates psychological and cardiovascular care. Building trust through empathetic communication, ensuring continuity of care, and minimizing retraumatization during medical encounters are key strategies. Incorporating mental health screening into cardiovascular clinics and providing education about the physical effects of PTSD can also facilitate earlier detection and management of CVD.

Evidence from intervention studies

Does Treating PTSD Reduce CVD Risk?

Emerging evidence suggests that effective treatment of PTSD may help reduce CVD risk by improving autonomic regulation, lowering inflammation, and promoting healthier behaviors. Although few studies have directly examined cardiovascular outcomes as primary endpoints, both psychotherapeutic and pharmacologic interventions for PTSD have shown benefits on physiological markers related to CVD risk.

Trauma-focused psychotherapy: Trauma-focused psychotherapies such as cognitive processing therapy and prolonged exposure are well-established first-line treatments for PTSD. These interventions substantially reduce PTSD symptom severity and are associated with improved autonomic balance and reduced physiological reactivity to stress [85]. Clinical research indicates that patients who respond to trauma-focused therapy demonstrate improved cardiovascular markers such as lower heart rate and blood pressure, suggesting reduced sympathetic activation [7]. Furthermore, evidence indicates that effective PTSD treatment may attenuate chronic stress responses that contribute to CVD development, particularly through normalization of autonomic and inflammatory pathways [3].

Selective serotonin reuptake inhibitors: SSRIs, including sertraline and paroxetine, are the most widely used pharmacologic treatments for PTSD. They alleviate re-experiencing, hyperarousal, and avoidance symptoms, thereby reducing chronic stress-related cardiovascular strain. Randomized controlled trials have confirmed the efficacy of sertraline in reducing PTSD symptom severity and improving overall functioning [86]. Beyond psychiatric improvement, SSRIs may positively influence cardiovascular physiology by reducing platelet aggregation, systemic inflammation, and resting heart rate, though evidence of direct CVD risk reduction remains mixed [87].

Prazosin for nightmares: Prazosin, an alpha-1 adrenergic receptor antagonist, is frequently prescribed for PTSD-related nightmares and sleep disturbances. By mitigating sympathetic hyperactivity and improving sleep quality, prazosin may reduce nocturnal elevations in blood pressure and heart rate [88]. Although large-scale randomized controlled trials have reported mixed effects on overall PTSD symptom improvement, prazosin remains beneficial for subgroups with prominent sleep disturbances, which are independently linked to increased cardiovascular risk [89].

CVD Prevention in PTSD

Cardiovascular disease prevention among individuals with PTSD is a growing area of clinical focus. Because PTSD is linked to autonomic imbalance, chronic inflammation, and unhealthy behaviors such as smoking and physical inactivity, targeted prevention strategies including exercise interventions, smoking cessation, and integrated care models are critical for reducing cardiovascular risk.

Exercise interventions: Physical activity is among the most effective non-pharmacological approaches for lowering CVD risk and improving psychological health. Exercise enhances autonomic regulation, reduces inflammation, and promotes vascular health. In patients with PTSD, aerobic exercise has been shown to reduce symptom severity while improving cardiorespiratory fitness, HRV, and blood pressure control [90]. A systematic review of exercise interventions in military veterans with PTSD reported consistent benefits in mood, sleep quality, and cardiovascular function, highlighting exercise as a valuable adjunctive therapy [91].

Smoking cessation programs: Smoking rates remain substantially higher in PTSD populations compared with the general public, significantly elevating CVD morbidity and mortality. Individuals with PTSD are more likely to smoke heavily and experience nicotine dependence and relapse [61]. Integrating trauma-informed psychotherapy with smoking cessation pharmacotherapy, such as varenicline or bupropion, has led to greater cessation success and improved adherence than standard programs [92]. Additionally, cognitive-behavioral interventions that simultaneously address PTSD symptoms and smoking triggers have demonstrated reduced relapse rates and improved abstinence [93].

Integrated care models: Care models that combine cardiovascular and mental health services are increasingly recognized as vital for holistic PTSD management. Coordinated care between cardiology, primary care, and psychiatry helps address both psychiatric symptoms and CVD risk factors, leading to better treatment engagement and clinical outcomes. Recent conceptual analyses and veteran-focused research have emphasized the importance of trauma-informed integrated care models that prioritize patient safety, empathy, and empowerment in medical settings [94]. Such approaches facilitate early detection of cardiovascular risk, improved adherence to medication, and better long-term management of comorbidities [41].

Clinical implications

Screening for CVD Risk in PTSD Patients

Screening for CVD risk among individuals with PTSD is a crucial component of comprehensive care. PTSD is independently associated with an increased risk of hypertension, CAD, stroke, and metabolic syndrome, even when controlling for traditional risk factors [3,95]. This elevated risk highlights the need for systematic cardiovascular assessment and preventive management within psychiatric and primary care settings.

PTSD patients frequently display physiological abnormalities such as elevated resting heart rate, blood pressure variability, and reduced HRV, all indicative of autonomic dysregulation [35]. Additionally, heightened systemic inflammation and altered HPA axis activity contribute to endothelial dysfunction and atherogenesis [9]. Comprehensive risk assessment should therefore encompass not only standard cardiovascular screening but also behavioral and psychosocial risk factors, including smoking, poor sleep, physical inactivity, and medication non-adherence.

Routine evaluation of lipid profiles, fasting glucose, glycated hemoglobin, and inflammatory markers such as CRP can help identify high-risk individuals early. Integrating cardiovascular risk assessment into PTSD management may facilitate timely interventions that reduce morbidity and mortality. Current literature emphasizes the importance of collaboration between mental health professionals, cardiologists, and primary care clinicians to provide holistic, trauma-informed care [3,41].

Validated tools such as the Framingham Risk Score, ASCVD Risk Estimator, and QRISK3 can be adapted for PTSD populations, though clinicians should account for the additional burden of psychological stress. Implementing trauma-informed practices during cardiovascular screening can also help reduce patient anxiety and avoidance, improving adherence to preventive care and follow-up appointments [41].

PTSD Screening in Cardiac Rehabilitation

Screening for PTSD within cardiac rehabilitation (CR) programs is an essential yet frequently overlooked component of comprehensive cardiovascular care. PTSD can develop following acute cardiac events such as MI, cardiac arrest, or invasive cardiac surgery and is associated with reduced adherence to rehabilitation, poor medication compliance, and higher risk of recurrent cardiovascular events [3,83]. Despite the established link between PTSD and adverse cardiovascular outcomes, routine screening in CR remains underutilized.

The prevalence of PTSD after major cardiac events ranges between 10% and 20%, with higher rates observed in patients who experience intense fear, helplessness, or perceived threat to life during their cardiac event or hospitalization [4]. PTSD symptoms such as intrusive thoughts, hyperarousal, and avoidance can hinder patient participation in exercise-based rehabilitation and discourage engagement in necessary medical follow-up [96]. Early detection of PTSD in CR settings is therefore vital to improving adherence, emotional recovery, and cardiovascular outcomes.

Validated tools such as the PTSD Checklist for DSM-5 (PCL-5), Primary Care PTSD Screen for DSM-5 (PC-PTSD-5), and the Impact of Event Scale-Revised (IES-R) have been effectively used in post-MI populations [97]. Incorporating such instruments during CR intake evaluations allows clinicians to identify patients who require trauma-informed psychological support. When integrated into multidisciplinary CR programs, early screening enables prompt referral for evidence-based treatments like cognitive processing therapy or SSRIs, improving both psychological and physiological recovery [98].

A trauma-informed CR model emphasizes empathy, trust, and collaboration between providers and patients, minimizing distress during clinical interactions and promoting adherence to exercise and medication regimens. Addressing PTSD symptoms in CR not only enhances emotional well-being but also contributes to improved cardiovascular regulation, reduced inflammation, and better long-term outcomes [41].

Multidisciplinary Care Models (Psychiatry + Cardiology)

Given the complex bidirectional relationship between PTSD and CVD, multidisciplinary care models integrating psychiatry and cardiology are increasingly recognized as effective approaches to improving outcomes. PTSD contributes to CVD through both biological mechanisms such as autonomic dysregulation, inflammation, and metabolic dysfunction, and behavioral factors including smoking, poor diet, and medication non-adherence [3,5]. Likewise, cardiovascular events and intensive medical interventions can precipitate PTSD, creating a cycle that requires coordinated, comprehensive care.

Integrated psychiatry and cardiology care: Traditional care models often treat PTSD and CVD separately, leading to fragmented management and suboptimal outcomes. Multidisciplinary models bridge this gap by fostering collaboration among psychiatrists, cardiologists, psychologists, and primary care providers. Such integration enables early detection of PTSD symptoms in cardiac patients and early cardiovascular screening among those with PTSD [83]. For example, integrating behavioral health services into cardiology clinics has been shown to improve medication adherence, engagement in cardiac rehabilitation, and reduce hospital readmissions [99].

Collaborative care interventions: Interventions that combine behavioral therapy, medication management, and cardiovascular monitoring have demonstrated effectiveness in improving both mental health and physical outcomes. In veterans, programs that combine PTSD therapy with cardiovascular risk management have been associated with better blood pressure control, lipid profiles, and psychological well-being [4]. Trauma-informed integrated care frameworks that emphasize empathy, safety, and shared decision-making (SDM) have been linked to greater patient satisfaction and treatment adherence [100].

Challenges and implementation: Despite strong evidence supporting multidisciplinary care, challenges remain. Barriers include limited access to mental health professionals within cardiology services, stigma associated with psychiatric care, and insufficient training among clinicians to identify trauma-related symptoms in cardiac patients. To address these issues, emerging research highlights the importance of integrating psychological and cardiovascular screening into high-stress professions and post-cardiac event care models to reduce long-term morbidity [100]. Integrated cardiovascular-behavioral medicine clinics represent promising solutions for the prevention and management of comorbid PTSD and CVD.

Shared Decision-Making and Trauma-Informed Cardiac Care

SDM and trauma-informed care (TIC) are essential frameworks for improving cardiovascular outcomes and patient satisfaction among individuals with PTSD. Traditional cardiac care often overlooks the psychological dimensions of illness and treatment, leading to avoidance, mistrust, and non-adherence in patients with trauma histories. Implementing SDM within a trauma-informed model allows clinicians to promote psychological safety, foster trust, and enhance engagement in cardiovascular care [41,101].

SDM in cardiovascular care: SDM emphasizes collaboration between patients and clinicians in choosing diagnostic and treatment options that align with the patient’s values, preferences, and context. This approach is particularly beneficial for patients with PTSD, whose autonomy and trust may be affected by prior traumatic experiences. Studies in cardiology show that SDM improves adherence, reduces decisional conflict, and enhances satisfaction with care [102]. In PTSD patients, SDM can also mitigate avoidance behaviors related to invasive procedures and encourage adherence to critical cardiovascular risk-reducing interventions such as exercise and medication compliance [103].

Trauma-informed care: TIC acknowledges the prevalence of trauma among cardiac patients and seeks to create a clinical environment that promotes safety, empowerment, and trust. The core principles of TIC include safety, transparency, collaboration, empowerment, and cultural sensitivity, which are especially relevant to patients whose cardiac events may themselves have been experienced as traumatic [104]. Evidence from both cardiovascular and behavioral medicine suggests that TIC reduces anxiety during procedures, improves rehabilitation participation, and enhances long-term adherence to medical recommendations [105].

Integrating SDM and TIC in clinical practice: Integrating SDM and TIC into cardiac care requires organizational changes and clinician training. Healthcare providers should recognize trauma-related responses such as avoidance, hyperarousal, or dissociation and adopt communication strategies that validate patient experiences. Structured collaboration, flexible pacing of consultations, and interdisciplinary care involving mental health professionals can strengthen both emotional and physical recovery [5]. To summarize the therapeutic evidence relevant to PTSD and cardiovascular risk, Table 4 outlines major intervention strategies, their physiological and psychiatric effects, and their potential cardiovascular implications.

**Table 4:** Interventions for PTSD and Their Potential Cardiovascular Implications

Intervention	Physiological Effect	Effect on PTSD Symptoms	Cardiovascular Relevance	Key References
Trauma-Focused Psychotherapy (CBT, PE, EMDR)	Reduces autonomic activation and sleep dysregulation	Large improvements in core PTSD symptom domains	Potential to lower BP, inflammation, and sympathetic arousal	Powers et al. [85]; Brady et al. [86]; Lespérance et al. [87]
SSRIs (e.g., sertraline, paroxetine)	Modulates serotonin signalling; improves sleep and mood regulation	Moderate–large improvement in PTSD severity	Potential indirect CVD benefit via stress reduction, sleep improvements, and adherence	Brady et al. [86]; Lespérance et al. [87]; Oh et al. [79]
Prazosin (α1-blocker)	Lowers sympathetic tone; improves night-time BP regulation	Effective reduction of nightmares and sleep disturbance in many populations	May reduce nocturnal BP variability and improve autonomic balance	Raskind et al. [88]; Raskind et al. [89]
Exercise & Structured Physical Activity	Improves metabolic profile and HRV; reduces inflammation	Reduces hyperarousal, improves sleep, depressive symptoms, and functioning	Direct cardiovascular benefit via cardiorespiratory fitness and metabolic risk control	Fetzner et al. [90]; Whitworth et al. [91]
Integrated/Collaborative Care Models	Improves treatment engagement, monitoring, and adherence	Reduction in PTSD burden, depression, and functional impairment	Improves risk-factor management and follow-up quality	Chambliss et al. [94]; Huffman et al. [99]
Mindfulness-Based Stress Reduction	Enhances parasympathetic tone and cortisol regulation	Reduces hyperarousal, anxiety, sleep problems, and avoidance	May improve BP, metabolic stress, and HRV	Sumner et al. [95]; Davidson et al. [96]
Sleep-Focused Interventions (CBT-I, OSA management)	Restores sleep architecture and reduces autonomic instability	Improves distress, nightmares, and daytime functioning	Evidence for BP lowering and arrhythmia reduction	von Känel et al. [97]; Shemesh et al. [98]

Future directions

Digital Health Interventions (Wearables, Telepsychiatry)

Digital health tools including wearable sensors, mobile applications, and telepsychiatry platforms are increasingly leveraged to target the overlapping domain of trauma‐related disorders such as PTSD and CVD). These technologies offer the promise of scalable, continuous monitoring and intervention, which may be particularly relevant given the shared behavioral and physiological pathways linking PTSD and CVD (e.g., autonomic dysregulation, sleep disturbance, physical inactivity).

Wearables and remote monitoring: Wearable devices can capture physiological signals (heart rate, HRV, electrodermal activity, movement, and sleep patterns) in real time, thus offering objective markers for both mental-health and cardiovascular risk states. For example, a cohort study using wrist-worn devices found that among individuals exposed to traumatic stress, wearable metrics of sleep and activity correlated with mental-health recovery markers [106]. Another feasibility pilot employed a “one-button tracker” wearable in psychotherapy with refugees diagnosed with complex PTSD; high user engagement was achieved and therapists reported enhanced insight into in-session and daily symptom dynamics [107]. In a cardiovascular context, wearables have been associated with increased health-care use and monitoring activity among atrial-fibrillation patients, suggesting potential for cross-pollination of trauma-cardio monitoring [108]. These findings suggest that wearables may serve dual functions: as early-warning systems for trauma reactivation and as adjuncts to cardiovascular risk-factor monitoring.

Telepsychiatry and mobile health applications: Telepsychiatry and digital behavioral-health apps enable remote delivery of psychological therapies (such as cognitive-behavioral therapy and exposure therapy) and self-monitoring tools, thereby improving access for trauma-exposed individuals who may have barriers to in-person care. A systematic review of telemedicine and e-health in CVD found that such platforms could reduce modifiable cardiovascular risk factors such as hypertension, obesity and inactivity [109]. Although specific trials combining PTSD-focused digital interventions with cardiovascular endpoints remain rare, the bridging of these fields is supported by preliminary evidence that digital mental-health interventions improve well-being and anxiety among users with self-reported cardiovascular risk factors [110]. Telepsychiatry therefore represents a viable modality for integrating trauma-care with cardiovascular prevention.

Integration of trauma-cardio digital models: Given the bidirectional relationship between PTSD and CVD, integrated digital interventions may offer added value. For instance, a model whereby a wearable continuously monitors autonomic or sleep signals, triggers prompts or telepsychiatry sessions when elevated arousal is detected, and simultaneously links to cardiovascular self-monitoring (e.g., blood pressure, activity), could enable synchronous trauma and cardiovascular risk management. Early proof-of-concept work supports the feasibility of such “hybrid” models though outcomes data on hard cardiovascular endpoints are lacking.

Barriers and implementation considerations: Key challenges include variability in digital-health literacy, device access and data privacy, as well as clinical integration of large volumes of sensor data. Wearable signals may reflect non-specific arousal states (exercise, excitement) rather than PTSD triggers, potentially limiting specificity [111]. Moreover, while digital platforms show promise for risk-factor improvement, it remains unclear whether they reduce major cardiovascular events among trauma-exposed populations. Cost-effectiveness, long-term adherence and equity of access must therefore be addressed before widespread implementation.

## Conclusions

PTSD is a significant and often underrecognized contributor to cardiovascular disease. The evidence reviewed demonstrates that chronic trauma-related stress affects multiple physiological systems, including autonomic regulation, the HPA axis, inflammatory pathways, endothelial function, coagulation, and metabolism. These biological disturbances work together with behavioral factors such as smoking, physical inactivity, poor diet, sleep disruption, and medication nonadherence to elevate cardiovascular risk. Clinical challenges including healthcare avoidance and delayed diagnosis further amplify poor outcomes.

Improving cardiovascular health in people with PTSD requires a comprehensive and trauma-informed approach. Effective PTSD treatment, structured exercise programs, sleep interventions, and integrated behavioral and cardiovascular care may help mitigate physiological strain and promote healthier behaviors. Routine cardiovascular risk screening in PTSD populations and targeted PTSD screening in cardiac care can support early detection and timely intervention. Continued research should explore whether treating PTSD directly improves cardiovascular outcomes and should evaluate new digital and multidisciplinary care models that address both conditions together.

## References

[1] Kessler RC, Sonnega A, Bromet E, Hughes M, Nelson CB (1995). Posttraumatic stress disorder in the National Comorbidity Survey. Arch Gen Psychiatry.

[2] Koenen KC, Ratanatharathorn A, Ng L (2017). Posttraumatic stress disorder in the World Mental Health Surveys. Psychol Med.

[3] Edmondson D, Cohen BE (2013). Posttraumatic stress disorder and cardiovascular disease. Prog Cardiovasc Dis.

[4] Coughlin SS (2011). Post-traumatic stress disorder and cardiovascular disease. Open Cardiovasc Med J.

[5] Padhi BK, Khatib MN, Serhan HA (2024). Cardiovascular impact of post-traumatic stress disorder: a systematic review and meta-analysis. Curr Probl Cardiol.

[6] Ebrahimi R, Dennis PA, Shroyer AL, Tseng CH, Alvarez CA, Beckham JC, Sumner JA (2024). Pathways linking post-traumatic stress disorder to incident ischemic heart disease in women: call to action. JACC Adv.

[7] Thayer JF, Friedman BH, Borkovec TD (1996). Autonomic characteristics of generalized anxiety disorder and worry. Biol Psychiatry.

[8] Lawrence S, Scofield RH (2024). Post traumatic stress disorder associated hypothalamic-pituitary-adrenal axis dysregulation and physical illness. Brain Behav Immun Health.

[9] Passos IC, Vasconcelos-Moreno MP, Costa LG (2015). Inflammatory markers in post-traumatic stress disorder: a systematic review, meta-analysis, and meta-regression. Lancet Psychiatry.

[10] Medina-Leyte DJ, Zepeda-García O, Domínguez-Pérez M, González-Garrido A, Villarreal-Molina T, Jacobo-Albavera L (2021). Endothelial dysfunction, inflammation and coronary artery disease: potential biomarkers and promising therapeutical approaches. Int J Mol Sci.

[11] Hoerster KD, Campbell S, Dolan M, Stappenbeck CA, Yard S, Simpson T, Nelson KM (2019). PTSD is associated with poor health behavior and greater body mass index through depression, increasing cardiovascular disease and diabetes risk among U.S. veterans. Prev Med Rep.

[12] Meinhausen C, Prather AA, Sumner JA (2022). Posttraumatic stress disorder (PTSD), sleep, and cardiovascular disease risk: a mechanism-focused narrative review. Health Psychol.

[13] Ginzburg K, Ein-Dor T, Solomon Z (2010). Comorbidity of posttraumatic stress disorder, anxiety and depression: a 20-year longitudinal study of war veterans. J Affect Disord.

[14] Gould F, Jones MT, Harvey PD (2021). The relationship between substance use, prior trauma history, and risk of developing post-traumatic stress disorder in the immediate aftermath of civilian trauma. J Psychiatr Res.

[15] Hitch C, Toner P, Armour C (2023). Enablers and barriers to military veterans seeking help for mental health and alcohol difficulties: a systematic review of the quantitative evidence. J Health Serv Res Policy.

[16] Varghese L, Emerson A (2022). Trauma-informed care in the primary care setting: an evolutionary analysis. J Am Assoc Nurse Pract.

[17] Singh A, Riaz R, Verma A (2025). Integrating mental health and cardiovascular wellness: synergistic impacts and the promise of comprehensive care models. Ann Med Surg (Lond).

[18] Ahmadi N, Hajsadeghi F, Mirshkarlo HB, Budoff M, Yehuda R, Ebrahimi R (2011). Post-traumatic stress disorder, coronary atherosclerosis, and mortality. Am J Cardiol.

[19] Edmondson D, Kronish IM, Shaffer JA, Falzon L, Burg MM (2013). Posttraumatic stress disorder and risk for coronary heart disease: a meta-analytic review. Am Heart J.

[20] Kinder LS, Bradley KA, Katon WJ, Ludman E, McDonell MB, Bryson CL (2008). Depression, posttraumatic stress disorder, and mortality. Psychosom Med.

[21] Falger PR, Op den Velde W, Hovens JE, Schouten EG, De Groen JH, Van Duijn H (1992). Current posttraumatic stress disorder and cardiovascular disease risk factors in Dutch Resistance veterans from World War II. Psychother Psychosom.

[22] Boscarino JA (1997). Diseases among men 20 years after exposure to severe stress: implications for clinical research and medical care. Psychosom Med.

[23] Crum-Cianflone NF, Bagnell ME, Schaller E (2014). Impact of combat deployment and posttraumatic stress disorder on newly reported coronary heart disease among US active duty and reserve forces. Circulation.

[24] Boscarino JA (2008). A prospective study of PTSD and early-age heart disease mortality among Vietnam veterans: implications for surveillance and prevention. Psychosom Med.

[25] Boscarino JA, Chang J (1999). Electrocardiogram abnormalities among men with stress-related psychiatric disorders: implications for coronary heart disease and clinical research. Ann Behav Med.

[26] Schnurr PP, Green BL (2004). Understanding relationships among trauma, post-traumatic stress disorders, and health outcomes. Trauma and Health: Physical Health Consequences of Exposure to Extreme Stress.

[27] Kang HK, Bullman TA, Taylor JW (2006). Risk of selected cardiovascular diseases and posttraumatic stress disorder among former World War II prisoners of war. Ann Epidemiol.

[28] Boscarino JA (2006). Posttraumatic stress disorder and mortality among U.S. Army veterans 30 years after military service. Ann Epidemiol.

[29] Kubzansky LD, Koenen KC, Spiro A 3rd, Vokonas PS, Sparrow D (2007). Prospective study of posttraumatic stress disorder symptoms and coronary heart disease in the Normative Aging Study. Arch Gen Psychiatry.

[30] Scherrer JF, Chrusciel T, Zeringue A (2010). Anxiety disorders increase risk for incident myocardial infarction in depressed and nondepressed Veterans Administration patients. Am Heart J.

[31] Beristianos MH, Yaffe K, Cohen B, Byers AL (2016). PTSD and risk of incident cardiovascular disease in aging veterans. Am J Geriatr Psychiatry.

[32] Rosman L, Sico JJ, Lampert R (2019). Posttraumatic stress disorder and risk for stroke in young and middle-aged adults: a 13-year cohort study. Stroke.

[33] El-Solh AA, Lawson Y, Attai P (2022). Cardiovascular events in insomnia patients with post-traumatic stress disorder. Sleep Med.

[34] Schneider M, Schwerdtfeger A (2020). Autonomic dysfunction in posttraumatic stress disorder indexed by heart rate variability: a meta-analysis. Psychol Med.

[35] Dennis PA, Dedert EA, Van Voorhees EE (2016). Examining the crux of autonomic dysfunction in posttraumatic stress disorder: whether chronic or situational distress underlies elevated heart rate and attenuated heart rate variability. Psychosom Med.

[36] Slavish DC, Ruggero CJ, Schuler K, Schwartz JE, Luft B, Kotov R (2024). Effects of daily posttraumatic stress disorder symptoms on heart rate variability. Psychosom Med.

[37] Park J, Marvar PJ, Liao P (2017). Baroreflex dysfunction and augmented sympathetic nerve responses during mental stress in veterans with post-traumatic stress disorder. J Physiol.

[38] Fonkoue IT, Marvar PJ, Norrholm SD (2018). Acute effects of device-guided slow breathing on sympathetic nerve activity and baroreflex sensitivity in posttraumatic stress disorder. Am J Physiol Heart Circ Physiol.

[39] Fonkoue IT, Hu Y, Jones T, Vemulapalli M, Sprick JD, Rothbaum B, Park J (2020). Eight weeks of device-guided slow breathing decreases sympathetic nervous reactivity to stress in posttraumatic stress disorder. Am J Physiol Regul Integr Comp Physiol.

[40] Yehuda R, Seckl J (2011). Minireview: stress-related psychiatric disorders with low cortisol levels: a metabolic hypothesis. Endocrinology.

[41] Yehuda R, Hoge CW, McFarlane AC (2015). Post-traumatic stress disorder. Nat Rev Dis Primers.

[42] Miller GE, Chen E, Zhou ES (2007). If it goes up, must it come down? Chronic stress and the hypothalamic-pituitary-adrenocortical axis in humans. Psychol Bull.

[43] Bremner JD, Licinio J, Darnell A (1997). Elevated CSF corticotropin-releasing factor concentrations in posttraumatic stress disorder. Am J Psychiatry.

[44] Dunlop BW, Wong A (2019). The hypothalamic-pituitary-adrenal axis in PTSD: Pathophysiology and treatment interventions. Prog Neuropsychopharmacol Biol Psychiatry.

[45] Katrinli S, Oliveira NC, Felger JC, Michopoulos V, Smith AK (2022). The role of the immune system in posttraumatic stress disorder. Transl Psychiatry.

[46] Yang JJ, Jiang W (2020). Immune biomarkers alterations in post-traumatic stress disorder: A systematic review and meta-analysis. J Affect Disord.

[47] Dong Y, Li S, Lu Y (2020). Stress-induced NLRP3 inflammasome activation negatively regulates fear memory in mice. J Neuroinflammation.

[48] Grenon SM, Owens CD, Alley H (2016). Posttraumatic stress disorder is associated with worse endothelial function among veterans. J Am Heart Assoc.

[49] Oosthuizen F, Wegener G, Harvey BH (2005). Nitric oxide as inflammatory mediator in post-traumatic stress disorder (PTSD): evidence from an animal model. Neuropsychiatr Dis Treat.

[50] Sher LD, Geddie H, Olivier L, Cairns M, Truter N, Beselaar L, Essop MF (2020). Chronic stress and endothelial dysfunction: mechanisms, experimental challenges, and the way ahead. Am J Physiol Heart Circ Physiol.

[51] Seligowski AV, Fonkoue IT, Noble NC (2022). Vagal control moderates the association between endothelial function and PTSD symptoms in women with T2DM. Brain Behav Immun Health.

[52] Vidović A, Grubišić-Ilić M, Kozarić-Kovačić D (2011). Exaggerated platelet reactivity to physiological agonists in war veterans with posttraumatic stress disorder. Psychoneuroendocrinology.

[53] Dietrich-Muszalska A, Wachowicz B (2017). Platelet haemostatic function in psychiatric disorders: Effects of antidepressants and antipsychotic drugs. World J Biol Psychiatry.

[54] von Känel R (2015). Acute mental stress and hemostasis: when physiology becomes vascular harm. Thromb Res.

[55] Robicsek O, Makhoul B, Klein E, Brenner B, Sarig G (2011). Hypercoagulation in chronic post-traumatic stress disorder. Isr Med Assoc J.

[56] Ahmadi N, Arora R, Vaidya N, Yehuda R, Ebrahimi R (2013). Post-traumatic stress disorder is associated with increased incidence of insulin resistance and metabolic syndrome. J Am Coll Cardiol.

[57] LIhua M, Tao Z, Hongbin M, Hui W, Caihong J, Xiaolian J (2020). Metabolic syndrome risk in relation to posttraumatic stress disorder among trauma-exposed civilians in Gansu Province, China. Medicine (Baltimore).

[58] Bharti V, Bhardwaj A, Elias DA, Metcalfe AW, Kim JS (2022). A systematic review and meta-analysis of lipid signatures in post-traumatic stress disorder. Front Psychiatry.

[59] Pericot-Valverde I, Elliott RJ, Miller ME, Tidey JW, Gaalema DE (2018). Posttraumatic stress disorder and tobacco use: a systematic review and meta-analysis. Addict Behav.

[60] Waldrop AE, Cohen BE (2014). Trauma exposure predicts alcohol, nicotine, and drug problems beyond the contribution of PTSD and depression in patients with cardiovascular disease: data from the Heart and Soul Study. Am J Addict.

[61] Fu SS, McFall M, Saxon AJ, Beckham JC, Carmody TP, Baker DG, Joseph AM (2007). Post-traumatic stress disorder and smoking: a systematic review. Nicotine Tob Res.

[62] Beckham JC, Dennis MF, McClernon FJ, Mozley SL, Collie CF, Vrana SR (2007). The effects of cigarette smoking on script-driven imagery in smokers with and without posttraumatic stress disorder. Addict Behav.

[63] Winning A, Gilsanz P, Koenen KC (2017). Post-traumatic stress disorder and 20-year physical activity trends among women. Am J Prev Med.

[64] Warburton DE, Bredin SS (2017). Health benefits of physical activity: a systematic review of current systematic reviews. Curr Opin Cardiol.

[65] Sumner JA, Cleveland S, Chen T, Gradus JL (2023). Psychological and biological mechanisms linking trauma with cardiovascular disease risk. Transl Psychiatry.

[66] van den Berk-Clark C, Secrest S, Walls J, Hallberg E, Lustman PJ, Schneider FD, Scherrer JF (2018). Association between posttraumatic stress disorder and lack of exercise, poor diet, obesity, and co-occuring smoking: a systematic review and meta-analysis. Health Psychol.

[67] Roer GE, Lien L, Bolstad I, Aaseth JO, Abebe DS (2023). The impact of PTSD on risk of cardiometabolic diseases: a national patient cohort study in Norway. BMC Psychiatry.

[68] Masodkar K, Johnson J, Peterson MJ (2016). A review of posttraumatic stress disorder and obesity: exploring the link. Prim Care Companion CNS Disord.

[69] Scherrer JF, Salas J, Lustman PJ (2018). The role of obesity in the association between posttraumatic stress disorder and incident diabetes. JAMA Psychiatry.

[70] So CJ, Miller KE, Gehrman PR (2023). Sleep disturbances associated with posttraumatic stress disorder. Psychiatr Ann.

[71] Koffel E, Khawaja IS, Germain A (2016). Sleep disturbances in posttraumatic stress disorder: updated review and implications for treatment. Psychiatr Ann.

[72] Wang L, Zhang S, Yu M, Yuan J (2022). Association between insomnia and subclinical atherosclerosis among Chinese steelworkers: a cross-sectional survey. Arch Public Health.

[73] Brownlow JA, Miller KE, Gehrman PR (2020). Treatment of sleep comorbidities in posttraumatic stress disorder. Curr Treat Options Psychiatry.

[74] Kronish IM, Edmondson D, Goldfinger JZ, Fei K, Horowitz CR (2012). Posttraumatic stress disorder and adherence to medications in survivors of strokes and transient ischemic attacks. Stroke.

[75] Gellad WF, Grenard JL, Marcum ZA (2011). A systematic review of barriers to medication adherence in the elderly: looking beyond cost and regimen complexity. Am J Geriatr Pharmacother.

[76] Kolandaivelu K, Leiden BB, O'Gara PT, Bhatt DL (2014). Non-adherence to cardiovascular medications. Eur Heart J.

[77] Yekehtaz H, Farokhnia M, Akhondzadeh S (2013). Cardiovascular considerations in antidepressant therapy: an evidence-based review. J Tehran Heart Cent.

[78] Castro VM, Clements CC, Murphy SN (2013). QT interval and antidepressant use: a cross sectional study of electronic health records. BMJ.

[79] Oh SW, Kim J, Myung SK, Hwang SS, Yoon DH (2014). Antidepressant use and risk of coronary heart disease: meta-analysis of observational studies. Br J Clin Pharmacol.

[80] De Hert M, Detraux J, van Winkel R, Yu W, Correll CU (2011). Metabolic and cardiovascular adverse effects associated with antipsychotic drugs. Nat Rev Endocrinol.

[81] Kung S, Espinel Z, Lapid MI (2012). Treatment of nightmares with prazosin: a systematic review. Mayo Clin Proc.

[82] Cohen BE, Edmondson D, Kronish IM (2015). State of the art review: depression, stress, anxiety, and cardiovascular disease. Am J Hypertens.

[83] Edmondson D, Richardson S, Falzon L, Davidson KW, Mills MA, Neria Y (2012). Posttraumatic stress disorder prevalence and risk of recurrence in acute coronary syndrome patients: a meta-analytic review. PLoS One.

[84] Browne KC, Chen JA, Hundt NE, Hudson TJ, Grubbs KM, Fortney JC (2021). Veterans self-reported reasons for non-attendance in psychotherapy for posttraumatic stress disorder. Psychol Serv.

[85] Powers MB, Halpern JM, Ferenschak MP, Gillihan SJ, Foa EB (2010). A meta-analytic review of prolonged exposure for posttraumatic stress disorder. Clin Psychol Rev.

[86] Brady K, Pearlstein T, Asnis GM, Baker D, Rothbaum B, Sikes CR, Farfel GM (2000). Efficacy and safety of sertraline treatment of posttraumatic stress disorder: a randomized controlled trial. JAMA.

[87] Lespérance F, Frasure-Smith N, Koszycki D (2007). Effects of citalopram and interpersonal psychotherapy on depression in patients with coronary artery disease: the Canadian Cardiac Randomized Evaluation of Antidepressant and Psychotherapy Efficacy (CREATE) trial. JAMA.

[88] Raskind MA, Peterson K, Williams T (2013). A trial of prazosin for combat trauma PTSD with nightmares in active-duty soldiers returned from Iraq and Afghanistan. Am J Psychiatry.

[89] Raskind MA, Peskind ER, Chow B (2018). Trial of prazosin for post-traumatic stress disorder in military veterans. N Engl J Med.

[90] Fetzner MG, Asmundson GJ (2015). Aerobic exercise reduces symptoms of posttraumatic stress disorder: a randomized controlled trial. Cogn Behav Ther.

[91] Whitworth JW, Ciccolo JT (2016). Exercise and post-traumatic stress disorder in military veterans: a systematic review. Mil Med.

[92] McFall M, Saxon AJ, Malte CA (2010). Integrating tobacco cessation into mental health care for posttraumatic stress disorder: a randomized controlled trial. JAMA.

[93] Dedert EA, Calhoun PS, Watkins LL, Sherwood A, Beckham JC (2010). Posttraumatic stress disorder, cardiovascular, and metabolic disease: a review of the evidence. Ann Behav Med.

[94] Chambliss T, Hsu JL, Chen ML (2024). Post-traumatic stress disorder in veterans: a concept analysis. Behav Sci (Basel).

[95] Sumner JA, Kubzansky LD, Elkind MS (2015). Trauma exposure and posttraumatic stress disorder symptoms predict onset of cardiovascular events in women. Circulation.

[96] Davidson KW, Burg MM, Kronish IM (2010). Association of anhedonia with recurrent major adverse cardiac events and mortality 1 year after acute coronary syndrome. Arch Gen Psychiatry.

[97] von Känel R, Hari R, Schmid JP (2011). Non-fatal cardiovascular outcome in patients with posttraumatic stress symptoms caused by myocardial infarction. J Cardiol.

[98] Shemesh E, Yehuda R, Milo O, Dinur I, Rudnick A, Vered Z, Cotter G (2004). Posttraumatic stress, nonadherence, and adverse outcome in survivors of a myocardial infarction. Psychosom Med.

[99] Huffman JC, Celano CM, Beach SR, Motiwala SR, Januzzi JL (2013). Depression and cardiac disease: epidemiology, mechanisms, and diagnosis. Cardiovasc Psychiatry Neurol.

[100] Angleman AJ, Van Hasselt VB, Schuhmann BB (2022). Relationship between posttraumatic stress symptoms and cardiovascular disease risk in firefighters. Behav Modif.

[101] Barry MJ, Edgman-Levitan S (2012). Shared decision making--pinnacle of patient-centered care. N Engl J Med.

[102] Joosten EA, DeFuentes-Merillas L, de Weert GH, Sensky T, van der Staak CP, de Jong CA (2008). Systematic review of the effects of shared decision-making on patient satisfaction, treatment adherence and health status. Psychother Psychosom.

[103] Elwyn G, Frosch D, Thomson R (2012). Shared decision making: a model for clinical practice. J Gen Intern Med.

[104] Raja S, Hasnain M, Hoersch M, Gove-Yin S, Rajagopalan C (2015). Trauma informed care in medicine: current knowledge and future research directions. Fam Community Health.

[105] McEwen BS, Gianaros PJ (2010). Central role of the brain in stress and adaptation: links to socioeconomic status, health, and disease. Ann N Y Acad Sci.

[106] Straus LD, An X, Ji Y (2023). Utility of wrist-wearable data for assessing pain, sleep, and anxiety outcomes after traumatic stress exposure. JAMA Psychiatry.

[107] Riisager LG, Huniche L, Larsen JE, Christiansen TB, Kring L, Palic S, Moeller SB (2025). Fostering engagement using a wearable for self-tracking assisted psychotherapy with refugees diagnosed with complex PTSD: a feasibility pilot study. Front Psychiatry.

[108] Rosman L, Lampert R, Zhuo S (2024). Wearable devices, health care use, and psychological well-being in patients with atrial fibrillation. J Am Heart Assoc.

[109] Jaén-Extremera J, Afanador-Restrepo DF, Rivas-Campo Y (2023). Effectiveness of telemedicine for reducing cardiovascular risk: a systematic review and meta-analysis. J Clin Med.

[110] Montgomery RM, Boucher EM, Honomichl RD (2021). The effects of a digital mental health intervention in adults with cardiovascular disease risk factors: analysis of real-world user data. JMIR Cardio.

[111] Kapogianni NA, Sideraki A, Anagnostopoulos CN (2025). Using smartwatches in stress management, mental health, and well-being: a systematic review. Algorithms.

